# *Harpagophytum procumbens* Root Extract Mediates Anti-Inflammatory Effects in Osteoarthritis Synoviocytes through CB2 Activation

**DOI:** 10.3390/ph15040457

**Published:** 2022-04-09

**Authors:** Alessia Mariano, Irene Bigioni, Roberto Mattioli, Antonella Di Sotto, Martina Leopizzi, Stefania Garzoli, Pier Francesco Mariani, Pietro Dalla Vedova, Sergio Ammendola, Anna Scotto d’Abusco

**Affiliations:** 1 Department of Biochemical Sciences, Sapienza University of Rome, 00185 Roma, Italy; alessia.mariano@uniroma1.it (A.M.); irene.bigioni@uniroma1.it (I.B.); roberto.mattioli@uniroma1.it (R.M.); 2 Department of Physiology and Pharmacology, Sapienza University of Rome, 00185 Rome, Italy; antonella.disotto@uniroma1.it; 3 Department of Medico-Surgical Sciences and Biotechnologies, Polo Pontino-Sapienza University, 04100 Latina, Italy; martina.leopizzi@uniroma1.it; 4 Department of Drug Chemistry and Technologies, Sapienza University of Rome, 00185 Rome, Italy; stefania.garzoli@uniroma1.it; 5 Casa di Cura Villa Stuart, 00135 Rome, Italy; pierfrancescomariani@pec.it; 6 UOC di Ortopedia e Traumatologia, Ospedale Santa Scolastica di Cassino, ASL di Frosinone, 03043 Cassino, Italy; pietro.dallavedova@aslfrosinone.it; 7 Ambiotec S.A.S., 04012 Cisterna di Latina (LT), Italy; ambiotec@libero.it

**Keywords:** osteoarthritis, fibroblast-like synoviocytes, endocannabinoid system, CB2, devil’s claw, harpagoside, volatile compounds, β-caryophyllene, eugenol, α-humulene

## Abstract

The endocannabinoid system is involved in the nociceptive and anti-inflammatory pathways, and a lowered expression of CB2 receptors has been associated with inflammatory conditions, such as osteoarthritis (OA). This suggests that CB2 modulators could be novel therapeutic tools to treat OA. In the present study, the involvement of *Harpagophytum procumbens* root extract, a common ingredient of nutraceuticals used to treat joint disorders, in CB2 modulation has been evaluated. Moreover, to clarify the effects of the pure single components, the bioactive constituent, harpagoside, and the main volatile compounds were studied alone or in a reconstituted mixture. Human fibroblast-like synoviocytes, extracted by joints of patients, who underwent a total knee replacement, were treated with an *H. procumbens* root extract dissolved in DMSO (HPE_DMSO_). The effectiveness of HPE_DMSO_ to affect CB2 pathways was studied by analyzing the modulation of cAMP, the activation of PKA and ERK MAP kinase, and the modulation of MMP-13 production. HPE_DMSO_ was able to inhibit the cAMP production and MAP kinase activation and to down-regulate the MMP-13 production. Pure compounds were less effective than the whole phytocomplex, thus suggesting the involvement of synergistic interactions. Present findings encourage further mechanistic studies and support the scientific basis of the use of *H. procumbens* in joint disorders.

## 1. Introduction

Osteoarthritis (OA) is a degenerative and inflammatory disease that affects the joints of the patients, causing pain and functional limitations. This is a pathology of the cartilage, a specialized tissue with a support function, which lubricates the articular surface and allows the sliding between the two joint heads. The degeneration of cartilage provokes strong friction of the subchondral bones in the joints, causing pain, swelling, and bone remodeling, in particular the formation of osteophytes. The joints mainly affected are those of the knees, hips, and fingers [1]. A growing body of evidence suggests that inflammation is strongly involved in OA pathogenesis [2]. Chemokines, cytokines, and other pro-inflammatory mediators have been found in synovial fluid, and systemic low-grade inflammation has been identified in OA patients [3]. In the synovium, two main types of synoviocytes are present, fibroblast-like synoviocytes (FLS) and synovial macrophages, which are both effectors of joint inflammation through the production of cytokines such as TNF-α and IL-6, chemokines such as IL-8 and matrix metalloproteases as MMP-13 [4,5]. All these factors are involved in the onset and progression of OA, inducing pain and irreversible cartilage degeneration. The early production of pro-inflammatory mediators from synoviocytes interferes with the resting state of chondrocytes, inducing these cells to produce pro-inflammatory cytokines themselves and shifting to a hypertrophic state, corresponding to the cartilage degeneration.

In the last years, the endocannabinoid system (ECS) has been described to be involved in the pain and inflammation in OA. ECS is based on two receptors, cannabinoid receptor type 1 (CB1) and cannabinoid receptor type 2 (CB2), which were first identified in the central nervous system and in immune cells, respectively [6]. Recently, the presence of CB2 in articular joints has been described both in chondrocytes and synoviocytes [7,8]. We previously described the presence of both CB1 and CB2 in synovial tissue, analyzing synovial membranes from OA and non-AO joint patients. We found that CB1 was moderately present and equally distributed in tissues from both OA and non-OA patients, whereas CB2 was expressed mainly in non-OA patients [9]. Moreover, CB2 has been described to mediate anti-inflammatory effects in synovial fibroblasts and macrophages [5]. Considering the anti-inflammatory role of CB2 in articular joints, therapies aimed at modulating CB2 activity could be considered a novel approach.

OA is an incurable disease, treated mainly with analgesic and anti-inflammatory agents, such as non-steroidal anti-inflammatory drugs, with the aim to alleviate symptoms [10], but they often show limited efficacy and multiple side effects. Several studies are underway with the aim of finding disease-modifying drugs, but to date, there are no approved drugs. Some molecules, such as glucosamine and glucosamine-derivative, have been studied [1,11,12], and some of them are administered as a nutraceutical in the long-term OA treatment [13], even though further studies are required to confirm their effectiveness. OA pain is also treated using traditional remedies, among them *Harpagophytum procumbens* (Burch.) DC. ex Meisn. (Fam. *Pedaliaceae*), commonly known as devil’s claw [14,15]. Studies have been conducted in OA animal models with the aim to evaluate the effects of *H. procumbens* root extracts on analgesic and anti-inflammatory pathways, showing that the whole extract had a dose-dependent activity, whereas the iridoid glycoside, harpagoside, a main component of the root was not effective [16]. On the other hand, other studies showed the effectiveness of harpagoside on osteoarthritis and low back pain [17]. Human clinical studies showed that the administration of *H. procumbens* root extract was able to attenuate the symptoms in OA patients [18,19,20]. The devil’s claw root contains several compounds other than harpagoside, among them phenolic glycosides, mono- and polysaccharides, triterpenes, phytosterols, flavonoids, and minor components such as volatile compounds [21]. The pharmacological activity is attributed to the whole phytocomplex, and few studies regarding the activity of the single *H. procumbens* root extract components are reported in the literature.

Previously, we studied the effects of *H. procumbens* root extract dissolved in aqueous or organic (DMSO) solvent in an in vitro model using human primary synoviocytes. We demonstrated that both extracts were able to stimulate the expression of CB2 both at the transcriptional and translational level and that HPE_DMSO_ was able to inhibit the Fatty Acid Anandamide Hydrolase (FAAH) activity [9]. Taking into account that CB2 is associated to G_i_ protein and thus its anti-inflammatory activity could be due to the effects on this pathway, here we analyzed whether the HPE_DMSO_ affects the intracellular signaling downstream of CB2. Moreover, to clarify the bioactive constituents and the possible interactions occurring under the phytocomplex, harpagoside, the biomarker of *H. procumbens* root extract and the main volatile compounds detected at the phytochemical analysis have been studied in comparison to the whole extract. 

## 2. Results

### 2.1. Effect of HPE_DMSO_ on cAMP Production

**Figure 1:**
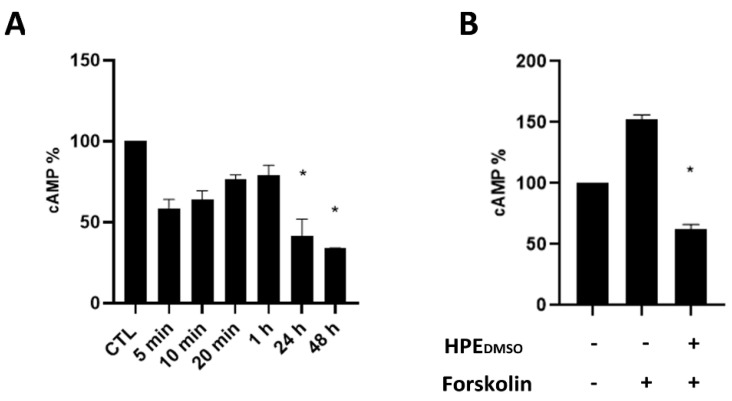
Determination of cAMP in cells treated with *H. procumbens* root extracted dissolved in DMSO (HPE_DMSO_). (**A**) Human primary synoviocytes (FLSs) were treated with 0.1 mg/mL of *Harpagophytum procumbens* root extract dissolved in DMSO (HPE_DMSO_), and after the indicated times, the amount of cAMP was determined by cAMP ELISA kit. (**B**) The amount of cAMP was determined in cells stimulated with forskolin for 20 min or in cells pre-treated with HPE_DMSO_ for 1 h and then stimulated with forskolin for 20 min. * *p* < 0.05 vs. CTL.

Previously, we found that the treatment with HPE_DMSO_ was able to induce the expression of cannabinoid receptor type 2 (CB2) in fibroblast-like synoviocytes (FLSs) [9]. In order to clarify the pathway that was affected by HPE_DMSO_ and considering that CB2 has been proven to be associated with Gα_i/0_, we evaluated the effects of this extract on the production of cAMP. Experiments revealed that a decrease in cAMP could be observed after 5 min of incubation, reaching a statistically significant difference only after 24 h up to 48 h of treatment (Figure 1A). The effect of HPE_DMSO_ on cAMP production was evident also after treatment with forskolin, which is a cAMP activator (Figure 1B).

### 2.2. Effect of HPE_DMSO_ on PKA Activation

**Figure 2:**
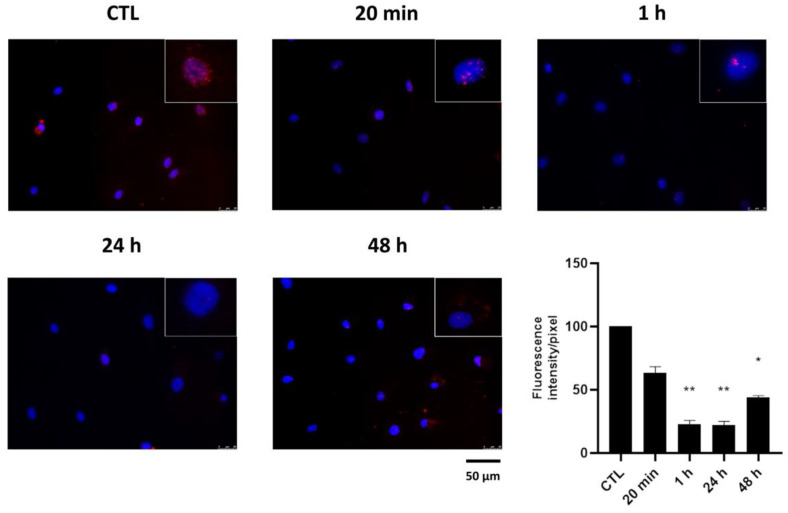
Effects of HPE_DMSO_ extract on PKA phosphorylation. Cells were treated with 0.1 mg/mL HPE_DMSO_ for the reported times and then analyzed by immunofluorescence using anti-*p*-PKA primary antibody and Alexa Fluor 568 (red) secondary antibody. Nuclei were stained with DAPI (original magnification 40×). The bar graph represents the pixel intensities in the region of interest, obtained by ImageJ. * *p* < 0.05; ** *p* < 0.01 vs. CTL.

**Figure 3:**
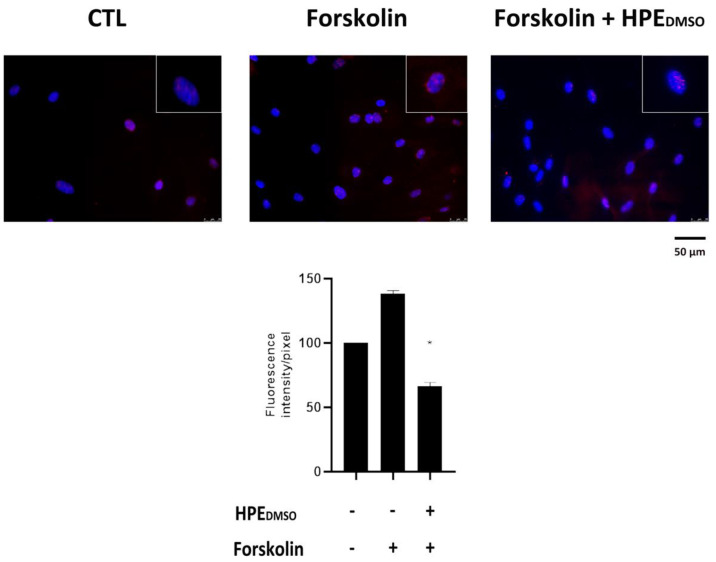
Effects of HPE_DMSO_ extract on PKA phosphorylation after forskolin stimulation. Cells were treated with forskolin for 30 min or pre-treated with HPE_DMSO_ for 1 h and then stimulated with forskolin for 30 min. Cells were analyzed by immunofluorescence using anti-*p*-PKA primary antibody and Alexa Fluor 568 (red) secondary antibody. Nuclei were stained with DAPI (original magnification 40×). The graph represents the pixel intensities in the region of interest, obtained by ImageJ. * *p* < 0.05 vs. CTL.

The decrease in cAMP affects the activation of PKA, which needs the up-regulation of cAMP to be released by its inhibitor and be activated through phosphorylation. In order to verify whether the extract exerted an effect on PKA, we analyzed the phosphorylation of PKA by immunofluorescence, finding that after treatment with HPE_DMSO_, the amount of *p*-PKA was decreased after 20 min and to a greater extent after 1 h, 24 h, and 48 h (Figure 2). Moreover, we analyzed the effect of HPE_DMSO_ after stimulation with forskolin, a cAMP activator, finding that the extract was able to decrease the amount of *p*-PKA, similarly to what was observed for cAMP in the previous experiment (Figure 3). These data confirm that the inhibition of PKA activation was a consequence of cAMP decrease. 

### 2.3. Effect of HPE_DMSO_ on ERK Phosphorylation 

**Figure 4:**
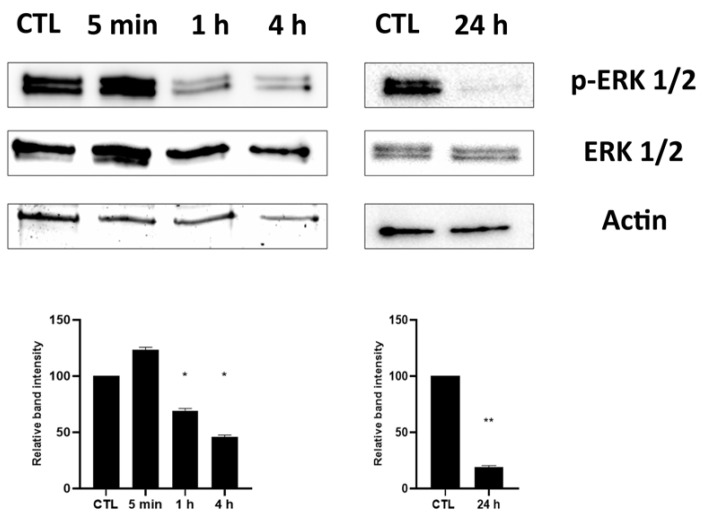
Effects of HPE_DMSO_ on ERK phosphorylation. Cells were left untreated (CTL) or treated with 0.1 mg/mL of HPE_DMSO_. Cell extracts were analyzed at 5 min, 1 h, 4 h and 24 h after treatment by western blot, using anti-ERK/*p*-ERK and actin antibodies (upper side). The densitometric analysis (bottom side) was performed by ImageJ. Results are expressed as mean ± SEM of data obtained by three independent experiments. * *p* < 0.05; ** *p* < 0.01 vs. CTL.

Among other effects, the decrease in PKA phosphorylation has an impact on MAPK activation. For this reason, we analyzed the effects of HPE_DMSO_ on ERK phosphorylation, finding a decrease in *p*-ERK, observed both at short times, 1 h and 4 h, and after 24 h exposure to HPE_DMSO_ (Figure 4).

### 2.4. Effect of HPE_DMSO_ on c-Fos and MMP-13 Expression

**Figure 5:**
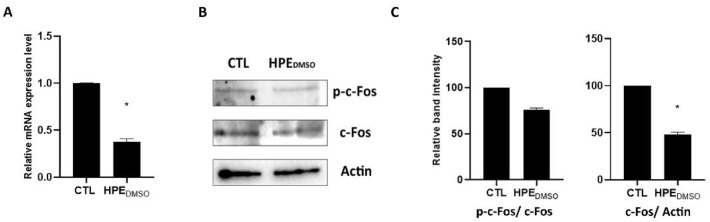
Effects of HPE_DMSO_ on c-Fos production and activation. (**A**) Cells were treated with 0.1 mg/mL HPE_DMSO_ for 24 h and then the mRNA was extracted and analyzed by RT-PCR. *c-Fos* mRNA level was reported as relative mRNA expression level with respect to 18S mRNA (2^−^^ΔΔCt^ method); (**B**) Cells were treated as described in **A** and then proteins were extracted and analyzed by western blot using anti-c-Fos/*p*-c-Fos and anti-actin antibodies; (**C**) Densitometric analysis of bands obtained in western blot experiment, performed by ImageJ. Results are expressed as mean ± SEM of data obtained by three independent experiments. * *p* < 0.05 vs. CTL.

The activation of *p*-ERK induces the activation of the transcription factor c-Fos; hence, we analyzed the expression level of *c-Fos* to verify whether the decrease in *p*-ERK was associated with a decrease in c-Fos. The treatment of cells with HPE_DMSO_ induced a statistically significant down-regulation of *c-Fos* mRNA expression level (Figure 5A). HPE_DMSO_ was also able to induce the decrease in c-Fos protein level but was unable to inhibit the activation of c-Fos, as shown by western blot experiments using both anti total c-Fos and anti *p*-c-Fos antibodies (Figure 5B, 5C).

**Figure 6:**
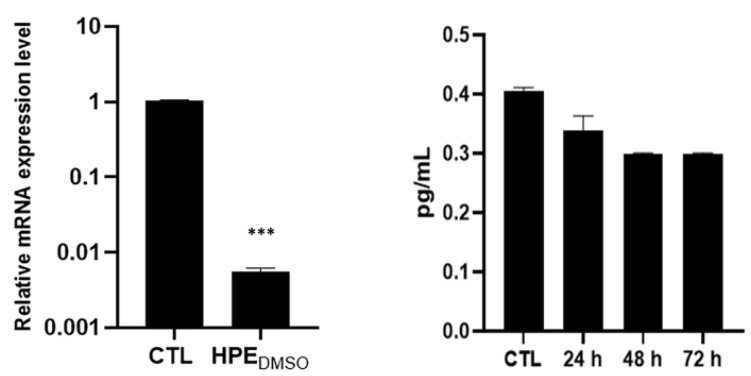
Effects of HPE_DMSO_ on MMP-13 production. Cells were treated with 0.1 mg/mL HPE_DMSO_ for 24 h and then the mRNA was extracted and analyzed by RT-PCR. MMP-13 mRNA level was reported as relative mRNA expression level with respect to 18S mRNA (2^−^^ΔΔCt^ method). The amount of MMP-13 produced was measured in the culture medium of cells and analyzed by ELISA. The results are reported as pg/mL. Results are expressed as mean ± SEM of data obtained by three independent experiments. *** *p* < 0.005 vs. CTL.

The up-regulation of metalloproteases, such as MMP-13, is a hallmark of osteoarthritis, where MMPs induce the degradation of extracellular matrix components. MMP-13 is under the control of AP-1 transcription factors, such as c-Fos. Considering that HPE_DMSO_ was able to down-regulate the production of c-Fos, we determined whether this extract had effects on MMP-13 production, finding that the treatment decreased both MMP-13 mRNA and protein levels, even if the down-regulation was statistically significant only at the transcriptional level (*** *p* > 0.005) (Figure 6).

### 2.5. Activity of Pure Compounds Contained in HPE_DMSO_ Alone and In Combination Compared to HPE_DMSO_

To investigate which was the contribution of the pure compounds detected by the phytochemical analysis to the activity of HPE_DMSO_, we treated the cells with β-caryophyllene, α-humulene, eugenol, and harpagoside. Taking into account the GC-MS analyses previously conducted in our laboratory, we chose the three volatile compounds more represented in the HPE_DMSO_ [9] and the harpagoside, which is considered the biomarker of *H. procumbens* root extract. The concentration of each compound was measured in 0.1 mg/mL of the HPE_DMSO_ (Table 1). We also assessed the effect of a mixture (Mix) containing β-caryophyllene, α-humulene, eugenol, and harpagoside at the same concentration found in 0.1 mg/mL HPE_DMSO._

**Figure 7:**
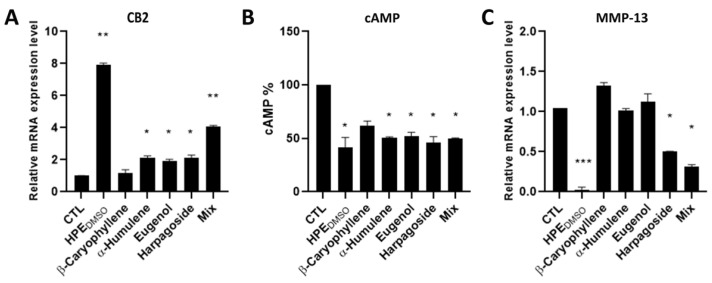
Effects of HPE_DMSO_ on CB2, cAMP and MMP-13 production. Cells were treated with 0.1 mg/mL HPE_DMSO_, 8 ng/mL β-caryophyllene, 2 ng/mL α-humulene, 0.4 ng/mL eugenol and 0.4 μg/mL harpagoside and with the mixture of all four compounds (Mix) at same concentration, for 24 h. After treatment, the mRNA was extracted and analyzed by RT-PCR. CB2 (**A**) and MMP-13 (**C**) mRNA level was reported as relative mRNA expression level with respect to 18S mRNA (2^−^^ΔΔCt^ method); The cAMP (**B**) amount was measured by cAMP ELISA kit. Results are expressed as mean ± SEM of data obtained by three independent experiments. * *p* < 0.05; ** *p* < 0.01; *** *p* < 0.005 vs. CTL.

Initially, we determined the effect of pure compounds on the mRNA expression level of the CB2 gene, finding that the expression was increased by all compounds except for β-caryophyllene, even if the α-humulene, eugenol, and harpagoside were found to be less efficacious than the mixture of all four compounds, and particularly less effective when compared to HPE_DMSO_ (Figure 7A). Then we determined the effects of the four isolated compounds on the inhibition of cAMP production. According to the results obtained on the CB2 mRNA expression level, all compounds, as well as the mixture, were able to decrease the cAMP production, even though β-caryophyllene decreased the cAMP in a non-statistically significant manner (Figure 7B). In order to evaluate the effects of the treatments on the MMP-13 production, we analyzed the mRNA expression level of this metalloprotease, finding that only the harpagoside and the mixture of the four components were able to decrease its production even if to a lower extent compared to HPE_DMSO_ (Figure 7C).

### 2.6. Effect of HPE_DMSO_ on Ca^2+^ Intracellular Level

**Figure 8:**
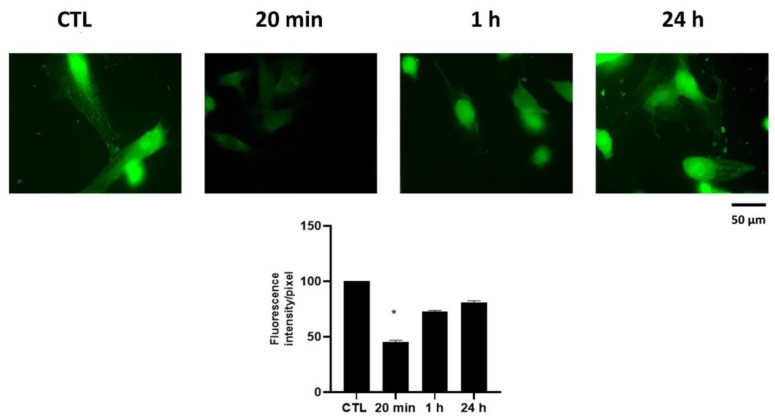
Effects of HPE_DMSO_ on intracellular Ca^2+^ concentration. Cells were treated with 0.1 mg/mL HPE_DMSO_ for 20 min, 1 h and 24 h, then the amount of intracellular Ca^2+^ concentration was measured by Calcium Green-1 AM. The densitometric analysis (bottom) was performed by ImageJ. Results are expressed as mean ± SEM of data obtained by three independent experiments. * *p* < 0.05 vs. CTL.

The activation of protein G_i/0_ also involves the activation of G_βγ_ subunits, which have been described to influence the calcium channel activity. We measured the amount of intracellular Ca^2+,^ finding that after 20 min HPE_DMSO_ treatment, it was drastically decreased (Figure 8). The Ca^2+^ amount returned back to the untreated cell level after 1 h, and it was stable over time. 

## 3. Discussion

Osteoarthritis is a degenerative joint disease characterized by chronic, low-grade inflammation, usually treated with anti-inflammatory drugs with the aim to relieve the symptoms [2,22]. The prolonged administration of these drugs leads to undesirable side effects; hence the search for symptomatic slow-acting drugs for osteoarthritis is strongly active. Nutraceuticals, such as glucosamine, chondroitin sulfate, and curcumin, are administered with the aim to delay cartilage destruction with inconsistent results [23]. In traditional medicine, plant extracts are administered to treat OA [15]; among them, the *H. procumbens* extract has been studied as a pro-inflammatory cytokine inhibitor [24]. The main components of *H. procumbens* extracts are the iridoid glycosides, among them the harpagoside, which is the most investigated one as a possible bioactive compound and is used as a marker for standardization purposes [14,25]. Some studies highlighted the anti-inflammatory effects of harpagoside through the inhibition of COX-1 and COX-2 enzymes and the down-regulation of pro-inflammatory cytokines and NO production [25]. Moreover, the effects of harpagoside have also been studied in human OA chondrocytes, where an anti-inflammatory activity has been described as a consequence of *c-Fos*/AP-1 signal suppression [20]. On the other hand, a study conducted in an animal model of OA showed analgesic and anti-inflammatory effects, whereas the purified harpagoside was not effective [16]. However, harpagoside was less effective as an anti-inflammatory agent when compared to *H. procumbens* root extracts, thus suggesting the involvement of other bioactive phytochemicals [14].

Several compounds have been identified in the *H. procumbens* root extract, among them the volatile compounds, such as β-caryophyllene, which are produced as a defense strategy to counteract pathogen and fungal infections of the plants [26,27]. In pre-clinical studies, β-caryophyllene, α-humulene, and eugenol were shown to have anti-inflammatory activity [28,29], and β-caryophyllene has been shown to act as an agonist of the CB2 receptor, which is involved in both the nociceptive and anti-inflammatory pathways [30,31]. Recently, this sesquiterpene has been shown to reduce articular and systemic inflammation in an animal model of arthritis through the mediation of crosstalk between CB2 and PPAR-γ receptors [32,33,34]. Previously, we showed that *H. procumbens* root extract, dissolved in water (HPE_H2O_) or in DMSO (HPE_DMSO_), was able to stimulate the production of CB2 both at mRNA and protein level, and we found that, in particular, HPE_DMSO_ contained an appreciable amount of volatile sesquiterpenes [9].

The aim of this study was to investigate the effects of HPE_DMSO_ on FLSs from OA patients, with regard to the pathways stimulated by CB2 and to reveal the contribution of the biomarker harpagoside and the main volatile compounds, β-caryophyllene, α-humulene and eugenol, to the CB2 pathway modulation by the whole phytocomplex. The choice to use HPE_DMSO_ was due to the ability of DMSO to recover most of the *H. procumbens* phytochemical, especially harpagoside and volatile compounds, and to retain the anti-inflammatory properties, as highlighted in our previous study. 

Regarding the in vitro model, we chose to utilize FLS, considering that in the articular joint, these cells support healthy chondrocytes but are also involved in the inflammatory processes during the onset and progression of OA. In OA, they produce pro-inflammatory cytokines that perturb the chondrocytes, leading these latter to produce pro-inflammatory mediators themselves and evolve toward a hypertrophic stage, which results in the disruption of articular cartilage [4,5]. In our previous study, we found that health synovial membranes expressed CB2, which was absent in OA synovial membranes [9]. This finding was in agreement with results obtained by Fukuda et al., who showed that CB2 was expressed on synovial membranes of Rheumatoid Arthritis (RA) patients and not in OA patients [35]. More recently, Rzeczycki et al. showed that the expression of CB2 is stimulated in the synovium of the OA mouse model as a protective response to damaged tissue [5]. The activation of CB2 in RA synoviocytes by CB2 agonist has been shown to block the production of pro-inflammatory mediators, inhibiting the adenylyl cyclase, thus decreasing the production of cAMP. The cAMP decrease blocked the activation of PKA, leading to the failure of activation of NF-κB [35]. Several studies showed that in OA joint, the activation of NF-κB is one of the main altered pathways causing the onset and progression of this pathology [12,36,37]. Thus, agents activating pathways able to inhibit NF-κB, can be considered suitable pharmacological strategies to treat OA. Previously, we found that the treatment of OA FLS with HPE_H2O_ and HPE_DMSO_ was able to stimulate the production of CB2 [9]. CB2 is associated with G_i_ protein, and its activation leads to the block of adenylyl cyclase, causing the down-regulation of the cAMP level. Treatment of the FLS with HPE_DMSO_ caused a borderline significant decrease in cAMP after 5 min treatment, whereas the cAMP decrease was statistically significant both at 24 h and 48 h, indicating a long-lasting effect of HPE_DMSO_ on G_i_ protein. Interestingly, HPE_DMSO_ was able to decrease cAMP also under stimulation of forskolin, and a diterpene was used to stimulate the adenylyl cyclase enzyme, thus confirming the activity of *H. procumbens* extract on this target. Accordingly, we found a decrease in *p*-PKA after 1 h treatment, which should be due to the decrease in cAMP at 5 min, and a decrease at 24 h and 48 h due to the down-regulation of cAMP at the same times. The activation of CB2 should induce the phosphorylation of ERK 1/2; hence we analyzed the effects of HPE_DMSO_ on the activation of this kinase, finding that *p*-ERK 1/2 decreased already after 1 h treatment up to 24 h. It may seem that this result is in disagreement with the activation of G_i_ protein, but it is in accordance with the results of Saroz and coworkers, who found that an inhibitor of the G_i/__βγ_ induced a decrease in *p*-ERK 1/2 [38]. Based on these observations, it could be speculated that HPE_DMSO_ stimulates G_αi_ and inhibits G_i/__βγ_.

As a consequence of the inhibition of *p*-ERK 1/2, we found a down-regulation of c-Fos production both at mRNA and protein levels. We did not observe a net inhibition of phosphorylation but a trend toward a decrease, most probably due to the decrease in c-Fos production. Finally, we verified whether there was a modulation of MMP-13, which is one of the metalloproteases more involved in the progression of OA along with MMP-1 and MMP-3 [39,40]. MMP-13 is under the control of c-Fos [41]; hence treatment of the cells with HPE_DMSO_ induced a decrease in this transcription factor and, in turn, a decrease in the MMP-13. In order to further verify whether the HPE_DMSO_ inhibited G_i/__βγ_ we checked the effect of this extract on intracellular Ca^2+^ level, finding that after 20 min treatment, Ca^2+^ was decreased, but at 1 h and 24 h, its level had returned to control levels. Considering that all effects we observed were always protracted, it could be hypothesized that the return to the initial level of Ca^2+^ could be due to the inhibition of G_i/__βγ_.

To clarify whether the results obtained with HPE_DMSO_ could be due to a specific compound in the whole extract or if possible synergistic interactions occurred, we evaluated the effects β-caryophyllene, α-humulene, eugenol, and harpagoside under our experimental conditions. Very interestingly, we found that β-caryophyllene, which is a CB2 receptor agonist, was not able to up-regulate CB2 receptors and consequently decreased the cAMP amount. Conversely, all other three analyzed compounds, i.e., α-humulene, eugenol, and harpagoside, were effective both in the up-regulation of the CB2 expression and in the decrease in cAMP. Regarding the modulation of MMP-13 metalloprotease, only the harpagoside was effective in decreasing its expression. Finally, we observed that the single isolated compounds were always less effective than HPE_DMSO_ and the mixture (Mix), even if at the same concentration found for HPE_DMSO_. Notably, the mixture was less effective than HPE_DMSO_; this evidence suggests that synergistic interactions among the analyzed compounds and other unknown molecules present in the phytocomplex should occur, each of them acting on different targets, contributing to the bioactivity of the whole extract. 

We can hypothesize that the observed effects of the whole extract are potentiated by the action of the single components. The CB2 up-regulation induced by α-humulene, eugenol, and harpagoside is supported by the activation of CB2 induced by β-caryophyllene and by the action of other compounds present in the *H. procumbens* root extract, which may also affect other pathways. Potentiating properties for both eugenol and caryophyllane sesquiterpenes, such as β-caryophyllene and α-humulene, have been highlighted when these molecules were administered in combination with anticancer drugs or herbal phytocomplexes [42,43]. Particularly, the synergic effects of caryophyllane sesquiterpenes have been found to be associated with the inhibition of efflux pumps, which leads to the intracellular accumulation of other substances [44]. Accordingly, three commercially available *H. procumbens* extracts have been shown to inhibit ABCB1/*p*-glycoprotein activity, despite a null effect of pure harpagoside [45]. This evidence strengthens our hypothesis about a possible potentiation of harpagoside anti-inflammatory effects within *H. procumbens* phytocomplex. 

Finally, it is noteworthy that these compounds and the whole phytocomplex act as phytoendocannabinoids affecting the receptor CB2 directly in the peripheral district, interfering with the nociception and the inflammation locally at the site of the injury. 

## 4. Materials and Methods

### 4.1. Human Primary Cell Isolation

Human primary fibroblast-like synoviocytes (FLSs) were isolated from synovial membranes, as previously described [9], from patients who underwent a total knee and hip arthroplasty. Full ethical consent was obtained from all donors, and the Research Ethics Committee, Sapienza University of Roma (#290/07, 29 March 2007), and ASL Lazio 2 (#005605/2019, 3 March 2019) approved the study. In brief, the minced synovial membrane fragments were treated with 1 mg/mL collagenase type IV and 0.25% trypsin for 1 h at 37 °C in agitation. Then, FLSs were cultured at 37 °C, and 5% CO_2_ in DMEM (Merck Life Science, Darmstadt, Germany) supplemented with l-glutamine, penicillin/streptomycin (Merck Life Science), and 10% Fetal Bovine Serum (FBS) and grown to 80% confluence. All experiments were carried out with synoviocytes at first passage (p1), isolated from at least 3 different donors.

### 4.2. Cell Treatment

Cells were left untreated (CTL) or treated, for the required time, as specified for each individual experiment, with 0.1 mg/mL of *Harpagophytum procumbens* root extract (HPE), provided by Ambiotec di Sergio Ammendola, and dissolved in DMSO (HPE_DMSO_). The concentration of 0.1 mg/mL was chosen based on the results obtained in our previous study [9].

In order to analyze the effect of single HPE_DMSO_ components on synoviocytes, cells were treated for 24 h with β-caryophyllene (Santa Cruz Biotechnology, Dallas, TX, USA, sc-251281A; ≥98% purity), α-humulene (Merck Life Science cod. 53675; ≥96% purity), eugenol (Merck Life Science, cod. W246700; ≥98% purity) and harpagoside (Merck Life Science, cod. 68527; ≥95% purity), according to the levels detected by phytochemical analysis in 0.1 mg/mL HPE_DMSO_ as described below. Moreover, we prepared a mixture containing all four components at concentrations determined in 0.1 mg/mL of HPE_DMSO_.

### 4.3. Measurement of cAMP

The amount of cAMP in untreated (CTL) and treated cells was measured by the Direct cyclic AMP Enzyme-Linked Immunosorbent Assay (ELISA) kit (Enzo Life Sciences, New York, NY, USA), according to the manufacturer’s instructions. Briefly, 10 × 10^4^ cells per well were seeded in 24-well plates and cultured for 5 min, 10 min, 20 min, 1 h, 24 h, and 48 h in presence of HPE_DMSO_. The effects of HPE_DMSO_ were also studied under the stimulation of forskolin (7-beta-Acetoxy-8, 13-epoxy-1a, 6b, 9atrihydroxy-labd-14-en-11-one), which is a potent, rapid, and reversible stimulator of adenylate cyclase activity and consequently an agent responsible of the cAMP increase. The cells were treated with forskolin for 20 min or pre-treated with the extract for 1 h and then stimulated with forskolin for 20 min [46].

### 4.4. Immunofluorescence

The activated PKA protein, p-PKA, was visualized by performing immunofluorescence experiments. A total of 10 × 10^3^ cells per well were seeded in 96-well plates and treated for 20 min, 1 h, 24 h, and 48 h in presence of HPE_DMSO_. In order to analyze the effects of HPE_DMSO_ under forskolin stimulation, the cells were treated with forskolin for 30 min or pre-treated with HPE_DMSO_ for 1 h and then stimulated with forskolin for 30 min. At the end of treatments, cells were washed in PBS, fixed in 100% ethanol for 15 min, and permeabilized with 0.5% Triton-X 100 in PBS for 10 min. After blocking with 3% bovine serum albumin (BSA) in PBS for 30 min, cells were incubated at 1 h with rabbit oligoclonal anti-*p*-PKA antibody (Invitrogen, Thermo Fisher Scientific, Waltham, MA, USA) 1:250. Cells were washed with PBS and then incubated for 1 h with Alexa Fluor 595 donkey anti-rabbit antibody 1:400 (Invitrogen, Thermo Fisher Scientific, to stain *p*-PKA in red. All steps were performed at room temperature. Slides were washed and then stained with DAPI (Invitrogen, Thermo Fisher Scientific) to visualize the nuclei. The images were captured by optical microscope Leica DM IL LED, using AF6000 modular Microscope (Leica Microsystem, Milan, Italy).

### 4.5. Western Blot Analysis

Western blot experiments were performed to analyze the effect of HPE_DMSO_ on phosphorylation of Extra-regulated kinase 1/2 (ERK 1/2). Cells, untreated and treated for 5 min, 1 h, 4 h, and 24 h with HPE_DMSO_, were washed with phosphate buffer saline (PBS) and lysated by protein extract kit (Active Motif, Carlsbad, CA, USA) in accordance with manufacturer’s instructions. Extracts were resolved on Mini-protean TGX precast gels (Bio-Rad Laboratories, Hercules, CA, USA) and transferred to PDVF membranes (Bio-Rad Laboratories) and probed with specific antibodies in accordance with the manufacturers’ instructions. Rabbit antibodies anti-ERK 1/2 and anti-*p*-ERK 1/2 (MyBioSource, San Diego, CA, USA) were used at 1:1000; rabbit antibodies anti-c-Fos and anti-*p*-c-Fos (Cell Signaling Technology, Danvers, MA, USA) at 1:500 and mouse anti-actin (Merck Life Science) at 1:1000. The secondary antibodies HRP-conjugate, both anti-rabbit and anti-mouse (Immunological Sciences, Rome, Italy) were used at 1:5000. The blots were revealed by ECL detection system (Advansta, Menlo Park, CA, USA). Image acquisitions were performed by ChemiDoc Instrument (Bio-Rad Laboratories). 

### 4.6. RNA Extraction and Reverse-Transcription

Total RNA was extracted from untreated and treated FLSs, with Blood/Tissues Total RNA extraction kit (Fisher Molecular Biology, Trevose, PA, USA), and the reverse transcription was performed according to the manufacturers’ instructions by Improm II enzyme (Promega Corporation, Madison, WI, USA).

### 4.7. Quantitative-Real Time-PCR 

Quantitative-Real Time-PCR analysis was performed using an ABI Prism 7300 (Applied Biosystems, Thermo Fisher Scientific, Waltham, MA, USA). Amplification was carried out using SensimixPlus SYBR Master mix (Bioline, London, United Kingdom). Primers (Table 2) synthesized by Biofab research (Rome, Italy), were designed using Primer Express software v1.4.0 (Applied Biosystems). Data were analyzed by 2^−ΔΔCt^ method, determining the transcript abundance relative to 18S housekeeping gene [47].

### 4.8. MMP-13 ELISA

The amount of MMP-13 in the cell supernatant was determined using Enzyme-Linked Immunosorbent assay kit (Fine Test ELISA, Fine Biotech Co., Ltd., Wuhan, China) according to the manufacturer’s instructions. Optical Density (O.D.) absorbance was measured at 450 nm by a microplate reader (NB-12-0035, NeBiotech, Holden, MA, USA).

### 4.9. Quantification of Chemical Constituents by GC–FID

The chemical composition of HPE_DMSO_ performed by SPME-GC/MS technique was described in our previously published paper [9]. β-caryophyllene, α-humulene, and eugenol, which were the principal volatile compounds detected in the extract, were quantified by Perkin Elmer Clarus 500 gas chromatography equipped with flame ionization detector (GC/FID) using the internal standard (nonane solution) method. The GC column was a Varian FactorFour VF-1 fused-silica capillary column (length 60 m × 0.32 mm ID × 1.0 μm film thickness). The oven temperature program was as follows: 60 °C for 2 min, then a gradient of 6 °C/min to 250 °C for 10 min, and the injector temperature of 270 °C. Injector and FID temperatures were set at 250 °C and 280 °C, respectively, and He was the carrier gas; data were processed by Perkin Elmer TotalChrom Navigator software. In order to calculate absolute quantitative data, the response factors for each compound were calculated by injections of pure analytical standards of β-caryophyllene, α-humulene, and eugenol at the concentration of 1 µg/mL.

### 4.10. UHPLC-MS Analyses

LC-MS determination of harpagoside concentration was performed on a Waters Acquity H-Class UHPLC system (Waters, Milford, MA, USA), including a quaternary solvent manager (QSM), a sample manager with flow-through needle system (FTN), a photodiode array detector (PDA) and a single-quadruple mass detector with electrospray ionization source (ACQUITY QDa). Chromatographic analyses were performed on a Kinetex C18 column (Phenomenex, 100 mm × 2.1 mm i.d., 2.6 μm particle size). Solvent A was 0.1% formic acid in water, and solvent B was 0.1% formic acid in CH_3_CN. Flow rate was set at 0.5 mL/min and column temperature at 25 °C.

Elution was performed isocratically for the first minute with 2% solvent B; from min 1 to min 6, solvent B was linearly increased to 55%, then, in 0.5 min, solvent B was set at 100% and maintained for 2 min. The column was re-equilibrated with 98% solvent A and 2% solvent B for 4 min before next injection. Harpagoside was identified by retention time, mass, and UV-Vis spectra, using a commercially available standard. The calibration curve was prepared as follows. Harpagoside at concentration of 0.01 mM was prepared in 0.1% aqueous formic acid/acetonitrile (95:5) (Merck Life Science), and then 0.5 μL, 1 μL, 2.5 μL and 5 μL were injected. HPE_DMSO_ at concentration of 0.1 mg/mL was prepared in the same solvent, and 10 μL or 20 μL were injected.

In the above-described chromatographic conditions, harpagoside has a retention time of ≅ 4.9 min. Mass spectrometric detection was performed in the negative electrospray ionization mode using nitrogen as nebulizer gas. Analyses were performed in Total Ion Current (TIC) mode in a mass range 100–1200 m/z. Capillary voltage was 0.8 kV, cone voltage 15 V, ion source temperature 120 °C, and probe temperature 600 °C. Calibration curves were generated with pure single compound, harpagoside (Sigma-Aldrich), by monitoring the H- ion (*m*/*z* 493.14).

### 4.11. Calcium Assay

The intracellular Ca^2+^ has been measured by Calcium Green-1 AM (Invitrogen, Thermo Fisher Scientific) following the manufacturer’s instructions, using 3 × 10^4^/cm^2^ cells treated with HPE_DMSO_ for 20 min, 1 h, and 24 h. The assay is based on the use of molecules that exhibit an increase in fluorescence upon binding Ca^2+^.

### 4.12. Densitometric Analyses

To perform the densitometric analysis of proteins production, the free software ImageJ v1.52t (https://imagej.nih.gov/ij/ downloaded on 1 July 2020) was used. For each cell culture condition, the integrated density values of chemiluminescence and fluorescence obtained in westerns blot and in immunofluorescence experiments, respectively, were considered.

### 4.13. Statistical Analysis

All data were obtained from at least three independent experiments, each performed either in duplicate or in triplicate. Data were statistically analyzed with two-way repeated-measures analysis of variance (ANOVA) with Bonferroni’s multiple comparison test, using Prism 5.0 software (GraphPad Software, San Diego, CA, USA). *p* value < 0.05 was considered significant.

## 5. Conclusions

The present results support the hypothesis that the anti-inflammatory effect of *H. procumbens* root extract is due to modulation of the endocannabinoid system and confirm that all the phytocomplex, with possible synergistic or additive interactions among iridoids and terpenes, especially caryophyllane sesquiterpenes, contribute to the observed effects. Alongside the anti-inflammatory and nociceptive effects, the stimulation of extracellular matrix components is very important in the treatment of OA. We are planning to study whether the HPE could also interfere with the anabolic metabolism of synoviocytes, as demonstrated in myofascial fibroblasts by Fede et al. [48]. Moreover, we are going to analyze the effects of *H. procumbens* root extract also on healthy human synoviocytes in order to verify whether the phenomena we observed in cells isolated from OA tissue also occur in healthy tissues.

Further studies are needed to characterize the suitable ratio between these classes of compounds to achieve the best healing effects and to fully outline the complex mechanisms involved.

## Figures and Tables

**Table 1:** Absolute concentration of major single volatile compounds and harpagoside, the main component of *H. procumbens* root extract. The concentrations were determined in 0.1 mg/mL HPE_DMSO._ Data are expressed as μg ± SEM per mg of extract (*n* = 3).

Compound	HPE_DMSO_ µg ±, SEM in 1 mg Extract
β-Caryophyllene	0.082 ± 0.0002
α-Humulene	0.024 ± 0.0004
Eugenol	0.004 ± 0.0002
Harpagoside	4 ± 0.0049

**Table 2:** Sequences of the primers used in RT-PCR analysis.

Gene	Primer Sequences
CB2 NM_009924	5′-ATGCTGTGCCTCATCAACTC-3′ 5′-CTCACACACTTCTTCCAGTG-3′
MMP-13 NM_002427	5′-TTCTTGTTGCTGCGCATGA-3′ 5′-TGCTCCAGGGTCCTTGGA-3′
c-FOS NM_005252	5′-CGAGCCCTTTGATGACTTCCT-3′ 5′-GGAGCGGGCTGTCTCAGA-3′
18S NM_003286.2	5′-CGCCGCTAGAGGTGAAATTC-3′ 5′-CATTCTTGGCAAATGCTTTCG-3′

## Data Availability

Data is contained within the article.
